# The Possibility of Reuse of Nanofiber Mats by Machine Washing at Different Temperatures

**DOI:** 10.3390/ma14174788

**Published:** 2021-08-24

**Authors:** Al Mamun, Imane Moulefera, Yusuf Topuz, Marah Trabelsi, Lilia Sabantina

**Affiliations:** 1 Junior Research Group “Nanomaterials”, Faculty of Engineering and Mathematics, Bielefeld University of Applied Sciences, 33619 Bielefeld, Germany; al.mamun@fh-bielefeld.de (A.M.); yusuf.topuz@fh-bielefeld.de (Y.T.); 2 Laboratory of Technique and Science of Water, University of Mascara Mustapha Stambouli, 29000 Mascara, Algeria; imanemoulefera@yahoo.fr; 3 Ecole Nationale d’Ingénieurs de Sfax, 3038 Sfax, Tunisia; marah.trabelsi@enis.tn

**Keywords:** COVID-19, nanofiber mats, machine washing of nanofiber mats, mask, sewability and ironing of nanofiber mats, reusable mask

## Abstract

The worldwide spread of coronavirus COVID-19 infections demonstrates the great need for personal protective equipment and, in particular, hygiene masks. These masks are essential for the primary protection of the respiratory tract against pathogens such as viruses and bacteria that are infectious and transmitted through the air as large droplets or via small airborne particles. The use of protective masks will continue to accompany humans for an indefinite period of time, and therefore there is an urgent need for a safe method to extend their usability by reusing them under perspective with minimal loss of protective properties. Nanofiber mats are widely used in masks and in this study the reusability of nanofiber mats is investigated by washing them at different temperatures. This paper shows the first measurements of the washability of nanofiber mats. Furthermore, the air permeability is measured, and the evaporation resistance is evaluated. According to the results of this study, the air permeability performance of nanofiber mats does not change significantly after washing, confirming the possibility of reuse.

## 1. Introduction

The coronavirus (COVID-19) pandemic is affecting 220 countries and territories. This increase in the contaminated has reached over 182.204.682 persons according to the World Health Organization (WHO). Due to this enormous number of infectious cases, the use of face masks is obligatory to protect human health. This health emergency is caused by the novel coronavirus, SARS-CoV-2, which is transmitted largely by the respiratory route (vide infra) [1,2].

Some scientists avow that COVID-19 cannot be transmitted while using the N95 (FFP2 in Europe) face mask, or, more pressing, that they decrease the percentage to which you are contagious. This is classified to be one of best face masks in filtration, but is limited to one-time use only [3,4]. Moreover, the filtering efficiency of protective face masks can be classified into single-use face masks, respirator masks, and surgical masks [5,6].

Electrospun nanofiber membranes are used in protective masks and provide physical barriers to particles and viruses through smaller fiber diameters and pore diameters; they remove the restriction of static electricity, in contrast to surgical masks [7]. Electrospinning technology provides an easy way to produce nanofiber mats [8,9] for various purposes, from a wide range of biobased and synthetic polymers [10,11,12] and admixtures of particles [13,14], etc. Using electrospinning technology, nanofiber mats can be produced easily and cost-effectively for use in electronics [15], battery storage [13,16], medical and biotechnology [17], water and air filtration [18], protective clothing [19,20], tissue engineering [21] and other applications.

Various research studies have proven that face masks can be reused after several steps such as washing using ultrasonic water bath, ozone convection, and a dry sterilization process with a regular steam process sterilization, etc. [22,23,24]. However, some limitations include inadequate filtration efficiency, their poor breathability, washability, and reusability to reinforce the applicability of these masks; their limitations are often further studied and overcome [25,26,27].

De Man et al. reported that a disposable surgical mask can be reused after correct sterilization. The sterilization is often undertaken using a dry sterilization process or regular steam process at 121 °C holding for five minutes, where the masks are placed in sealed bags [24]. Juang and Tsai suggested cleaning the N95 mask with boiling water for five minutes [28]. Wang et al. have suggested soaking medical, surgical, and N95 masks in hot water at a temperature greater than 56 °C for 30 min, based on the method for killing the COVID-19 virus proposed by the National Health Commission of the People’s Republic of China [29,30]. These processes are considered to be an efficient method to sterilize face masks by inactivating the virus due to the high temperature applied. In addition, earlier research proved the performance of this method through blind comparison of a secondhand mask with an unused mask of permeability properties, pressure drop, and filtration capacity. The results demonstrate the high efficacy of the process which leads to the same properties as unused masks, even after multiple sterilizations [31]. Hence, dry sterilization and a daily steam process are often a great choice to affect the shortage of masks in hospitals [26]. Additionally, current studies have been analyzing the sterilization of N95 filter-based face masks using UV light as another method for the reusability of the face masks [32]. As mentioned the N95 filter is manufactured by a melt-blown (MB) process. Many researchers have indicated that the N95 from MB display an efficient filtration of below 80%, which can be related to the fabric structures [4].

Li et al. also reported another treatment that used the nanoparticles as nanofunctional materials in the N95 and surgical masks. They suggested that the wearing of nanotreated surgical masks show significantly lower heart rates [33]. Moreover, in vivo tests also reported the same method and they prove to have an additional protective functions in stopping capillary diffusion and antibacterial activities in both the N95 and surgical masks [34]. In this sense, the reuse of face masks can be a solution to many peoples by investigating a new and easy treatment method, applied at home with simple products. As indicated in [4], the nanofiber MB face masks have the same properties as unusable masks by spraying and dipping treatments using 75% ethanol for evaluation of reusability [34].

Recently, the production of electrospun nanofibers increase due to the wide range of applications as in healthcare [35,36,37,38], environmental engineering [39,40] and energy storage sectors [41]. Nanofibers are the simplest replacement for microfibers and thin films thanks to their different properties, such as high surface area which may be functionalized for the desired property, their morphology, and more importantly the easy technique for production of nanofibrous mats.

All of these proprieties have caused nanofiber filters to be gradually used for mask applications across the world. As reported in previous work, nanofiber mats can be used as filters in many applications, as well as in the environmental sector [17].

All of the studies mentioned above require special chemicals, solvents or special devices for the decontamination of masks. This proposed study examines the reusability of nanofiber mats by suggesting a simple and inexpensive method. The nanofiber mats are washed at different temperatures in a household washing machine and examined, which forms the focus for this study. The air permeability is measured, and the evaporation resistance is evaluated to check the effectiveness of these properties after the washing process at different temperatures. Many methods have been proposed by scientists to decontaminate the masks and to increase the reuse of the masks in order to reduce the environmental impact of microplastic [42,43]. According to our research and to the best of our knowledge, no studies were found about the washability of nanofiber mats in a washing machine, which is the focus of this study. This study offers a simple and cost-effective way to remove contamination by washing. In addition, only one study was found, which was conducted by our research group, regarding the sewability of nanofiber mats [44]. Furthermore, no studies were found where the ironing of nanofiber mats is concerned. This study should provide an insight into the simple method of decontamination of nanofiber mats that could be part of the masks, using household washing machines and ironing, with the aim of increasing reusability, reducing microplastic fibers and thus protecting the environment. To overcome the scarcity of masks in the future, this study shows that reuse of the mask is possible and it could make a contribution to circumvent the scarcity of masks.

## 2. Materials and Methods

Electrospun nanofiber mats were produced using an electrospinning machine (Elmarco Ltd., Liberec, Czech Republic) and Polypropylene (PP) as a non-woven substrate machine (Elmarco Ltd., Liberec, Czech Republic) for collecting the nanofibers. For the fabrication of nanofiber mats the following spinning parameters were used: high voltage of 80 kV, nozzle diameter 0.9 mm, distance lower electrode/substrate 240 mm, distance lower electrode/substrate 50 mm, carriage speed 100 mm/s, temperature in the chamber 24 °C, relative humidity of 33%. The duration of electrospinning was 15 min.

The electrospinning solution contained 16 wt% polyacrylonitrile (PAN) (X-PAN (Dralon GmbH, Lingen, Germany) and 84% dimethyl sulfoxide (DMSO) (min. 99.9%, obtained from S3 Chemicals, Bad Oeynhausen, Germany). The reason for using DMSO as a solvent was because of its low-toxic properties [26,27]. The polymer solution was stirred using a magnetic stirrer at room temperature for 2 h.

Investigations of material chemistry and nanofiber morphology were performed using a Fourier transform infrared (FTIR) spectrometer, Excalibur 3100 (Varian, Inc., Palo Alto, CA, USA). For optical evaluations of the nanofiber mats a confocal laser scanning microscope (CLSM) VHX-600K (Keyence, Neu-Isenburg, Germany) was used.

A Permetest skin model (Sensora Textile Measuring Instruments and Consulting, Liberec, Czech Republic) was used to measure the water vapor resistance. The measurements were performed in three areas per sample.

Air permeability was applied using the machine (Textest AG, Testing Instruments for Quality Control, FX 3300 Lab Air, Zürich, Switzerland) with a test area of 20 cm^2^ and test pressure of 100 Pa.

The double-lockstitch sewing machine (Dürkopp Adler GmbH, Bielefed, Germany) with bottom feed and needle system 134 and needle gauge 70 (Groz-Beckert KG, Albstadt, Germany) was used for the sewing work.

The 100% polyester sewing thread (No. 120) (Gütermann GmbH, Gutach-Breisgau, Germany) was used for the sewing tests.

The ironing machine (Veit Varioset S+B/Veit GmbH, Landsberg/Lech, Germany) was used for the ironing of the samples, applying a temperature of 180 °C with steam for 5 s and without steam.

For washing tests, a household washing machine (Miele Softtronic W439, Gütersloh, Germany) was used.

**Figure 1:**
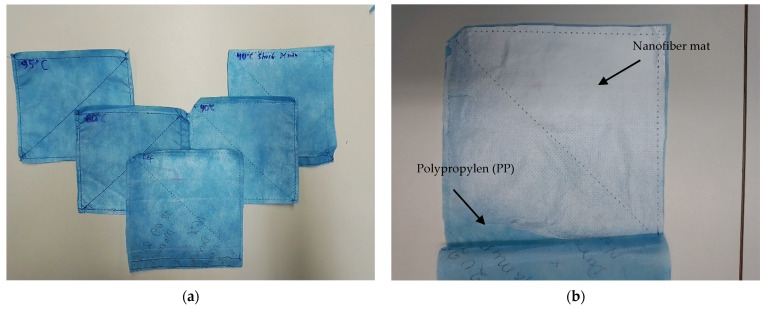
Overview of the samples before washing test (**a**) and a reference sample after washing test (PP/PAN nanofiber mat/PP composite) (**b**).

The preparation of samples was performed as follows: first, the nanofiber mat was placed between the PP non-woven, and by means of the sewing machine the square shapes were sewn through all three layers, forming PP/nanofiber mat/PP composites. After that, the samples were cut and had the final size of 15 cm × 15 cm. The samples were sewn through in the middle for a better fixation of all layers during the washing process. Figure 1 shows the overview of the samples and the composition of the layers in the sample.

After preparing the samples they were washed at 40 °C, 40 °C (short program of 21 min), 60 °C and 95 °C temperature, respectively, by a household washing machine without a spin cycle and without additional protection such as a laundry bag. The washed samples were then compared with a non-washed sample to determine the differences. The overview of the samples and washing parameters is given in Table 1.

The samples were washed at defined temperatures (see Table 1) and washing parameters and then dried in the air at room temperature for 24 h.

The ironing tests were performed on nanofiber mats with the temperature at 180 °C with steam and without steam for 5 s. For further investigation, the seams of PP/PAN nanofiber mat/PP composite were unraveled (see Figure 1b).

Filtration efficiency of the samples was tested using a TSI model 8130A (TSI GmbH, Aachen, Germany) automatic filter tester with a 10 cm diameter pressure plate. The standard sample holder of the 8130A was used. The test method was related to DIN EN 143:2007. Aerosol generation was performed according to specifications and standards with an oil aerosol generator model S/N 72027005 with the test flow rate of 95 l/min. For the oil aerosol specifications, a poly-alpha-olefin (PAO) paraffin oil aerosol with a mass mean diameter of 0.4 µm and a count median diameter of 0.16 µm was selected for validation of the specific filter efficiency for respirators according to EN 143. Aerosol particle size and geometric standard deviation were determined using a TSI Model 3934 Scanning Mobility Particle Sizer.

## 3. Results and Discussion

**Figure 2:**
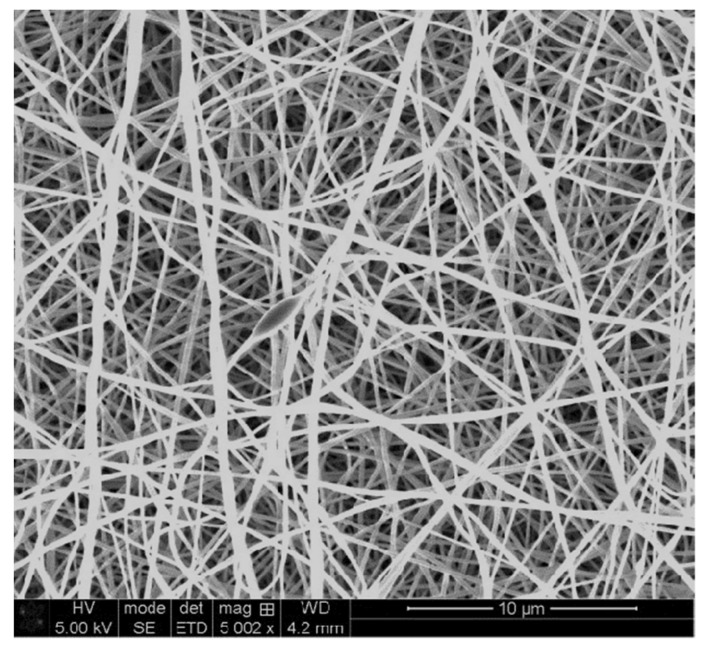
Scanning Electron Microscope (SEM) image of 16% PAN nanofiber mat [45]. Reproduced using a Creative Commons Attribution (CC BY 4.0) license.

Figure 2 shows the SEM image of PAN nanofiber mat, which was also used in this study as well as our previous study [45]. As shown here, the nanofibers are relatively straight and almost bead free.

**Figure 3:**
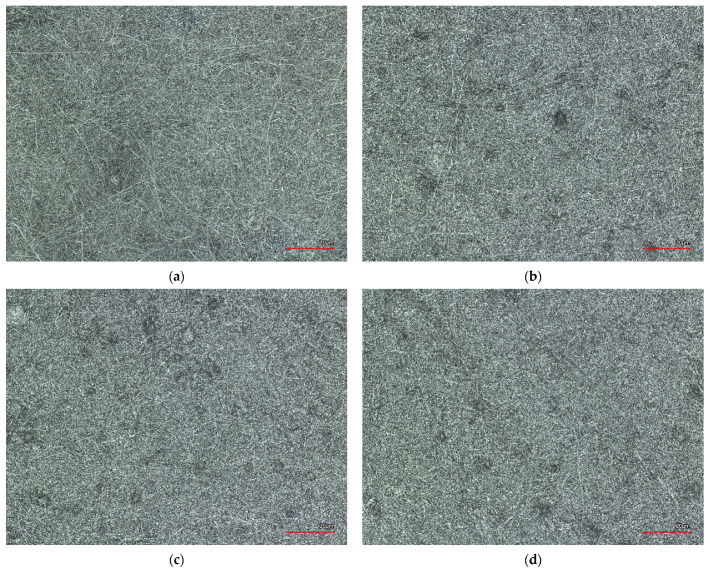

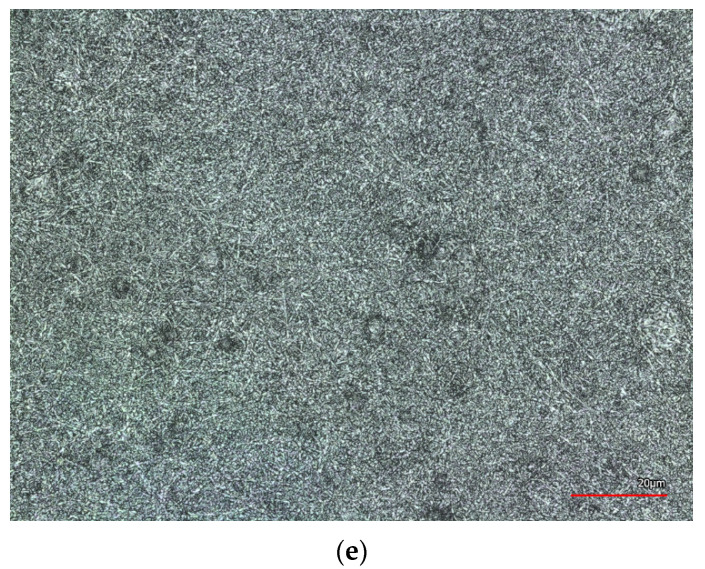
Confocal Laser Scanning Microscope (CLSM) images of (**a**) a PAN nanofiber mat before the washing process; (**b**) washed at 40 °C (short program); (**c**) washed at 40 °C; (**d**) washed at 60 °C and (**e**) washed at 95 °C. The scale bars indicate 20 μm.

Confocal Laser Scanning Microscope (CLSM) images in Figure 3 show the nanofiber mats after washing at 40 °C (short program), 40 °C, 60 °C and 95 °C temperatures.

As can be seen in the CLSM images in Figure 3, the washing tests did not cause any visible changes to the surface of the nanofiber mats and the images do not differ visually from each other. Only a slight densification of the nanofibers could be observed, probably because the PAN nanofiber mats were washed at different temperatures. In the unwashed nanofiber mate (1a), some thicker nanofiber mats are visible on the surface, and after the washing tests they are less visible.

**Figure 4:**
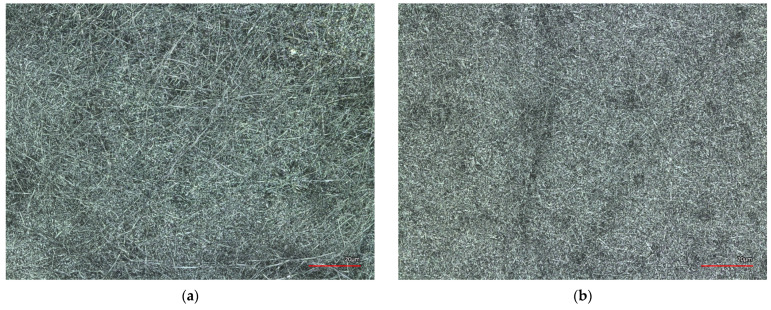
Confocal Laser Scanning Microscope (CLSM) images of nanofiber mats ironed (**a**) at 180 °C without steam and (**b**) at 180 °C using steam for 5 s. The scale bars indicate 20 μm.

This could be because the melting temperature of PAN is 300 °C. At temperatures higher than 150 °C, cyclisation of the polymer occurs, and PAN nanofiber mats start to change color, which is well known from previous studies [46,47]. The white nanofiber mats change color from white to yellow and dark brown at the oxidative stabilization at 280 °C and become almost black during carbonization at 800 °C [48]. Polyacrylonitrile (PAN) nanofiber mats are usually used as a precursor for carbon nanofiber production [49]. After the ironing process, the nanofiber mat did not change color, probably because the temperature exposure time in the ironing process was too short and the temperature was not high enough. The color change of the nanofiber mat in the CLSM images appears visible and, according to our previous studies, should be in a range from a light brown to dark brown color, which is not the case here [45,48] (see Figure 4b).

In addition, a slight densification of the nanofibers in Figure 4b was observed after the ironing process, presumably because the PAN nanofiber mats contract and shrink in size when exposed to temperature. This effect is well known from previous studies when oxidative stabilization of a nanofiber mat occurs before the carbonization process [45,48,49]. In the non-ironed nanofiber mat (Figure 4a), thicker nanofiber mats can be seen on the surface which are not as visible after the ironing test (Figure 4b). Presumably, the surface morphology has changed due to the factors described above.

**Figure 5:**
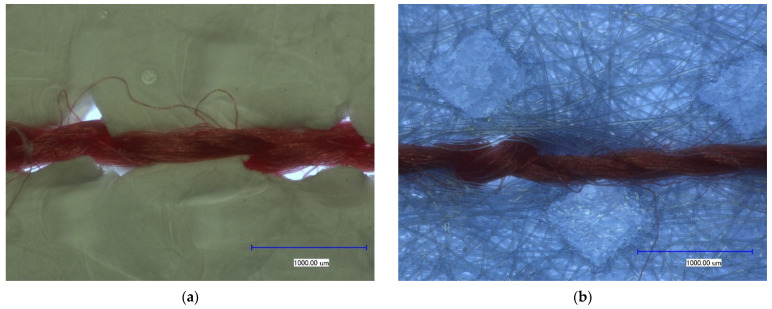
Microscopic images of the sewing tests of (**a**) a nanofiber mat and (**b**) PP.

Figure 5 shows the seam on the nanofiber mat and PP. For a better contrast here the sewing thread was used in the color red (see Figure 5).

Here, it could be found that the sewing needle damaged the nanofiber mat and the holes of insertion point of the sewing needle is clearly visible. This means that the sewing of layers of nanofibers involves damage as well as weakening of the seams.

**Figure 6:**
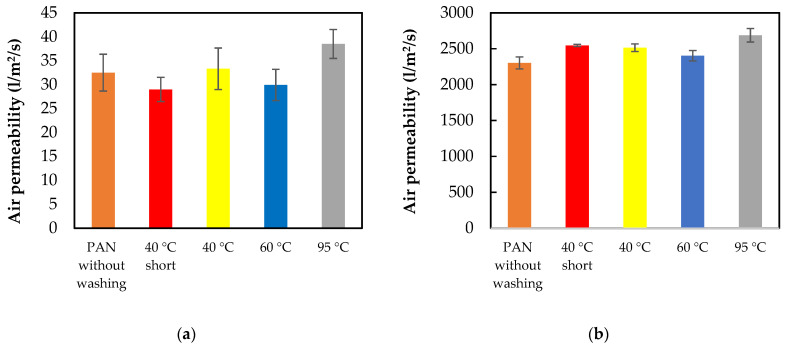
Air permeability of (**a**) nanofiber mats and (**b**) PP (Polypropylene).

To analyze the reusability of nanofiber mats and PP, washing tests were performed and air permeability was tested after washing tests. The air permeability of the nanofiber mats and PP is shown in Figure 6.

Interestingly, nanofiber mats show almost the same air permeability before the washing test and after washing at 60 °C. Additionally, the samples washed at 40 °C (short program) and 60 °C show almost the same results. The highest air permeability was measured in samples washed at 95 °C.

For PP samples, the tendency is similar to nanofiber mats, and only a small difference can be seen in the PP washed at 40 °C (short program). In this case, the air permeability is higher compared to nanofiber mats. The air permeability results of nanofiber mats are shown in Table 2.

The air permeability results of PP are shown in Table 3.

As expected, the difference between air permeability results of nanofiber mats are in a maximum range of (33.3 ± 4.3) l/m^2^/s and have a PP of (2688 ± 94) l/m^2^/s. The difference in air permeability between the nanofiber mat and PP consists of the different fiber diameters, thickness, porosity and the different surface morphology. The PP fibers are in the macro range and the nanofiber in the nano range, and therefore they have different properties.

**Figure 7:**
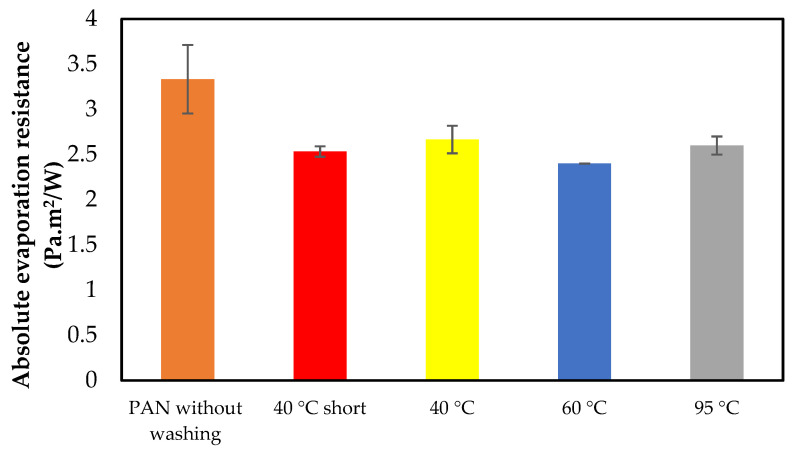
Absolute evaporation resistance of nanofiber mats.

Figure 7 displays the absolute evaporation resistance of washed nanofiber mats.

It can be seen that the evaporation resistance decreases with the washing treatment and also by increasing the washing temperature from 40 °C to 95 °C. This can be observed by a reference sample (without washing treatment) showing (3.33 ± 0.37) Pa·m^2^/W and changing to (2.60 ± 0.10) Pa·m^2^/W for the sample washed at 95 °C. Interestingly, samples washed at 40 °C and 95 °C show a slight increase in evaporate resistance compared to samples washed at 40 °C (short program) and 60 °C.

The absolute evaporation results of nanofiber mats are shown in Table 4.

**Figure 8:**
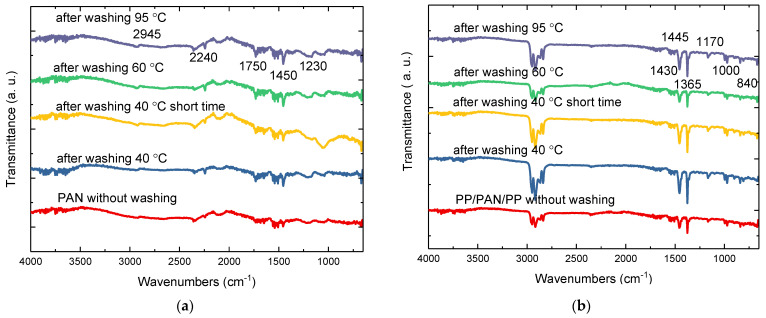
FTIR spectra of (**a**) PAN nanofiber mats and (**b**) PP/PAN nanofiber mat/PP composite.

Figure 8a,b show the characteristic FTIR peaks of PAN nanofiber mats and PP/PAN nanofiber mat/PP composite (reference samples) without washing and after washing at 40 °C (short program), 40 °C, 60 °C and at 95 °C, respectively. Here, the influence of the mat morphology, which presumably changed during the washing process, on the water vapor permeability through PAN nanofiber mats is shown (see Figure 7 and Table 4). At this point it can be stated that the washing procedures have an influence on air permeability and evaporation resistance. According to Figure 6 and Table 2 and Table 3, as the washing temperature rises, the air permeability of nanofiber mats and PP/PAN nanofiber mat/PP composite increases, which is probably due to changes in the surface morphology due to the effect of the washing process. In addition, the evaporation resistance also increases after the washing attempts and when the temperature is increased. In summary, it can be assumed that the washing procedure has no negative influence on the air permeability and evaporation resistance of the nanofiber mats, and that the nanofiber mats can be washed. The surface morphology of the nanofiber mats changes as a result of the washing treatment, but not in so relevant a way that it has a negative impact.

In Figure 8a, it can be seen that there is no significant difference between all spectra after washing PAN nanofiber mats at different temperatures. However, it shows large differences between the peaks before and after washing the nanofiber mats. Additionally, in Figure 8b, it can be seen that there is no significant difference between the spectra after washed PP/PAN nanofiber mat/PP composite at different temperatures, and only the difference from washed PP to unwashed samples is visible.

The Fourier transform infrared spectroscopy (FTIR) analysis method was used for the analysis of chemical composition and interactions by scattering of infrared waves through the material. The FTIR spectra of the main functional group of PAN include the frequency range of peaks at 2240 cm^−1^, indicating the nitrile group (C≡N), and 1740–1750 cm^−1^, assigned to the C=O group. Characteristic stretching frequencies for are C≡C (frequency range of 1230 cm^−1^), -CH_3_ symmetric (peak at 1450 cm^−1^ and 2945 cm^−1^) [47]. The typical PAN peaks are not as pronounced probably because the nanofiber mats thickness affected the FTIR measurement results.

The FTIR spectrum of the PP/PAN nanofiber mat/PP composite (Figure 8b) shows absorption peaks of polypropylene (PP) such as moderate absorption peaks of deformation vibrations of the plane methylene group in the spectral range of 1445 to 1485 cm^−1^ as well methyl groups in range of 1430–1470 cm^−1^ or 1365 to 1395 cm^−1^. Moreover, a peak at 2915 m^−1^ is attributed to vibrations of CH bands. The peaks at 840 cm^−1^, 1000 cm^−1^, and 1170 cm^−1^ are vibrations of terminal unsaturated CH_2_ groups [50].

The filtration efficiencies of the three-layer (PP/PAN nanofiber mat/PP) and two-layer (PP/PAN nanofiber mat) samples were exemplarily tested.

The three-layer samples (PP/PAN nanofiber mat/PP composite) were sewn in the middle for better hold during the washing tests (see Figure 1). Despite the holes at the puncture points of the sewing needle, a high filter efficiency was observed. The unwashed samples show 93.86% filter efficiency and washed samples show 86.07% (washed at 60 °C) and 88.60% (washed at 95 °C), respectively. The two-layer samples (PP/nanofiber mat composite) without sewing tests show 99.98 % filter efficiency. It could be stated here that the unwashed masks show a high efficiency that is reduced when washed. At this point, there is also a need for future research to further specify the results.

## 4. Conclusions

The problem with today’s masks is that they are used only once and then disposed of, polluting the environment and nature. The world’s daily need for masks is enormous, and the nature of single use exacerbates the scarcity of masks and the waste of scarce resources. Meanwhile, there are some proposals for disinfection of these masks, which alleviates the shortage situation of the masks; however, they are not always easy and require special equipment and facilities or the use of chemicals. In this study, the household washing machine approach to nanofiber mat decontamination can be offered as a simple and inexpensive method without the use of chemicals or additional solvents and special equipment. Compared to other decontamination methods with the use of ozone or organic solvents, water vapor, ultraviolet (UV) radiation or hydrogen peroxide vapor [51], decontamination with hot water and using the washing machine is an effective method. With this study we have shown that it is possible to wash the nanofiber mats and reuse them. It was found that the sewn-through samples kept the filtering efficiency of the masks with nanofiber mat relatively high, confirming the usefulness of nanofibers as protection. Future research at this site is needed to further specify these results. These results open a long-term perspective of the use of nanofiber mats in functional masks. This reusability of masks can contribute to solving the dilemma of mask shortage worldwide. The reuse will extend the lifetime of the masks while maintaining filtration efficiency and secondary infection by viral residues can thus be prevented.

## Figures and Tables

**Table 1:** Overview of the washing parameters of the samples.

Washing Temperature (°C)	Washing Process Duration (Time)
40 °C	21 min
40 °C	75 min
60 °C	75 min
95 °C	75 min

**Table 2:** The air permeability measurement results of nanofiber mats.

Samples	Air Permeability Mean Value (l/m^2^/s)	Standard Deviation (SD)
PAN nanofiber mat without washing	32.5	±3.9
40 °C short program	29.0	±2.5
40 °C	33.3	±4.3
60 °C	29.9	±3.3
95 °C	38.5	±3.0

**Table 3:** The air permeability measurement results of PP.

Samples	Air Permeability Mean Value (l/m^2^/s)	Standard Deviation (SD)
PAN without washing	2302	±83
40 °C short program	2546	±16
40 °C	2514	±52
60 °C	2402	±72
95 °C	2688	±94

**Table 4:** The absolute evaporation resistance of nanofiber mats.

Samples	Absolute Evaporation Resistance Mean Value (Pa·m^2^/W)	Standard Deviation (SD)
PAN without washing	3.33	±0.37
40 °C short program	2.53	±0.05
40 °C	2.67	±0.15
60 °C	2.40	±0.10
95 °C	2.60	±0.10

## Data Availability

The data created in this study are fully depicted in the article.
